# Recent tobacco smoking, restaurant and in-car secondhand smoke exposure are associated with depressive symptoms among young adults: a population-based cross-sectional analysis

**DOI:** 10.1038/s41598-024-54575-y

**Published:** 2024-03-04

**Authors:** Hongfei Mo, Changhong Wang, Yan Li

**Affiliations:** 1 Synergetic Innovation Center of Kinesis and Health, School of Physical Education (Main Campus), Zhengzhou University, Zhengzhou, Henan People’s Republic of China; 2 College of Public Health, Zhengzhou University, Zhengzhou, Henan People’s Republic of China; 3 The Second Affiliated Hospital of Xinxiang Medical University, Xinxiang, People’s Republic of China; 4 The Second Affiliated Hospital of Zhengzhou University, Zhengzhou, Henan People’s Republic of China

**Keywords:** Depressive symptoms, Recent tobacco smoking, Secondhand smoke exposure, Young adults, Health care, Health occupations, Medical research, Rheumatology

## Abstract

The purpose of this study was to evaluate the association between recent tobacco smoking, household secondhand smoke exposure, confined space secondhand smoke exposure and depressive symptoms in young adults after adjustments for each other. Data from NHANES 2013–2018 were extracted. A total of 4129 young adults age 18–35 years (mean age 26.11 ± 5.39 years, 2021 males and 2108 females) were included. Depressive symptoms were screened by PHQ-9. Recent tobacco smoking was assessed through question “smoked tobacco in the last 5 days?”. Household secondhand smoke exposure was assessed through question “living with a smoker who smoked inside the house?”. Confined space secondhand smoke exposure was assessed by SSEQ. Binary logistic regression models were performed to analyze the associations. Significant association were observed in recent tobacco smoking (OR = 1.593, 95% CI 1.318–1.926) and confined space secondhand smoke exposure (OR = 1.399, 95% CI 1.185–1.651), but not in household secondhand smoke exposure (*P* = 0.108). Among the different settings of confined space secondhand smoke exposure, restaurant (OR = 1.732, 95% CI 1.120–2.678) and in-car (OR = 1.350, 95% CI 1.102–1.652) exposure were significantly associated with depressive symptom after after fully adjustments.

## Introduction

Early adulthood can be a challenging stage of life. The transition from adolescence to adulthood is characterized by a variety of social adaptations^1^, such as changes in social networks, relationships, and roles. Young adults who have just reached adulthood are particularly vulnerable to adverse reactions to these changes, which can lead to the development of depressive symptoms^2^.

Depressive symptoms are key features of major depressive disorder and other related mental health conditions. They encompass a range of emotional, cognitive, behavioral, and physical manifestations that significantly impact an individual's mood, thoughts, and daily functioning. Common depressive symptoms include persistent sadness, loss of interest or pleasure in activities, changes in appetite or weight, sleep disturbances, fatigue, feelings of guilt or worthlessness, difficulty concentrating, and recurrent thoughts of death or suicide^3^. These symptom can be debilitating and can severely impact multiple aspects of a young adult's life, including school performance, social life, and overall well-being^4^. Evidence suggests that depression in late adolescence is a significant predictor of suicide in early adulthood^5^.

The consequences of depressive symptoms can be particularly severe in young adults as they are facing the challenges of transitioning from adolescence to adulthood. Unfortunately, however, depressive symptoms in young adults are currently in the process of worsening and pose a serious public health burden. Studies have shown that the Covid-19 pandemic had also more significantly exacerbated depressive conditions in young adults^6^.

Previous studies suggest that smoking and secondhand smoke exposure in confined spaces may significantly affect depressive symptoms in young adults. Results from a Korean epidemiological study showed that smoking behavior significantly increased the risk of depressive symptoms in young adults, while exposure to secondhand smoke in the home and indoor environment is also identified as a potential risk factor^7^. These findings are supported by another study conducted in Germany and China^8^. Active smoking played a similar role to passive secondhand smoke exposure in terms of toxicant exposure. It was found that nicotine from cigarette smoke may directly affect the neurotransmitter system, leading to changes in mood and behavior^9^. Another study suggests that exposure to secondhand smoke may lead to chronic inflammation and oxidative stress, which may negatively impact mental health^10^. In addition, household exposure may be more likely to lead to depressive symptoms in young people than secondhand smoke exposure in other settings because such exposures are often chronic and ongoing, difficult to avoid or escape from, and children or young people are more vulnerable.

There are multiple potential causes of depressive symptoms in young adults, including genetic factors, social factors, environmental factors, health conditions, growing stress and chemical imbalances in the brain. These factors can interact in complex ways to trigger or exacerbate depressive symptoms, making it challenging to identify effective interventions and supportive policies^11^. Therefore, we have adjusted for multiple covariates that may influence depressive symptom in our study to seek more precise results. The purpose of this study was to evaluate whether recent tobacco smoking, household secondhand smoke exposure and confined space secondhand smoke exposure are associated with depressive symptom in young adults after adjustments for each other.

## Materials and methods

### Study population

**Figure 1:**
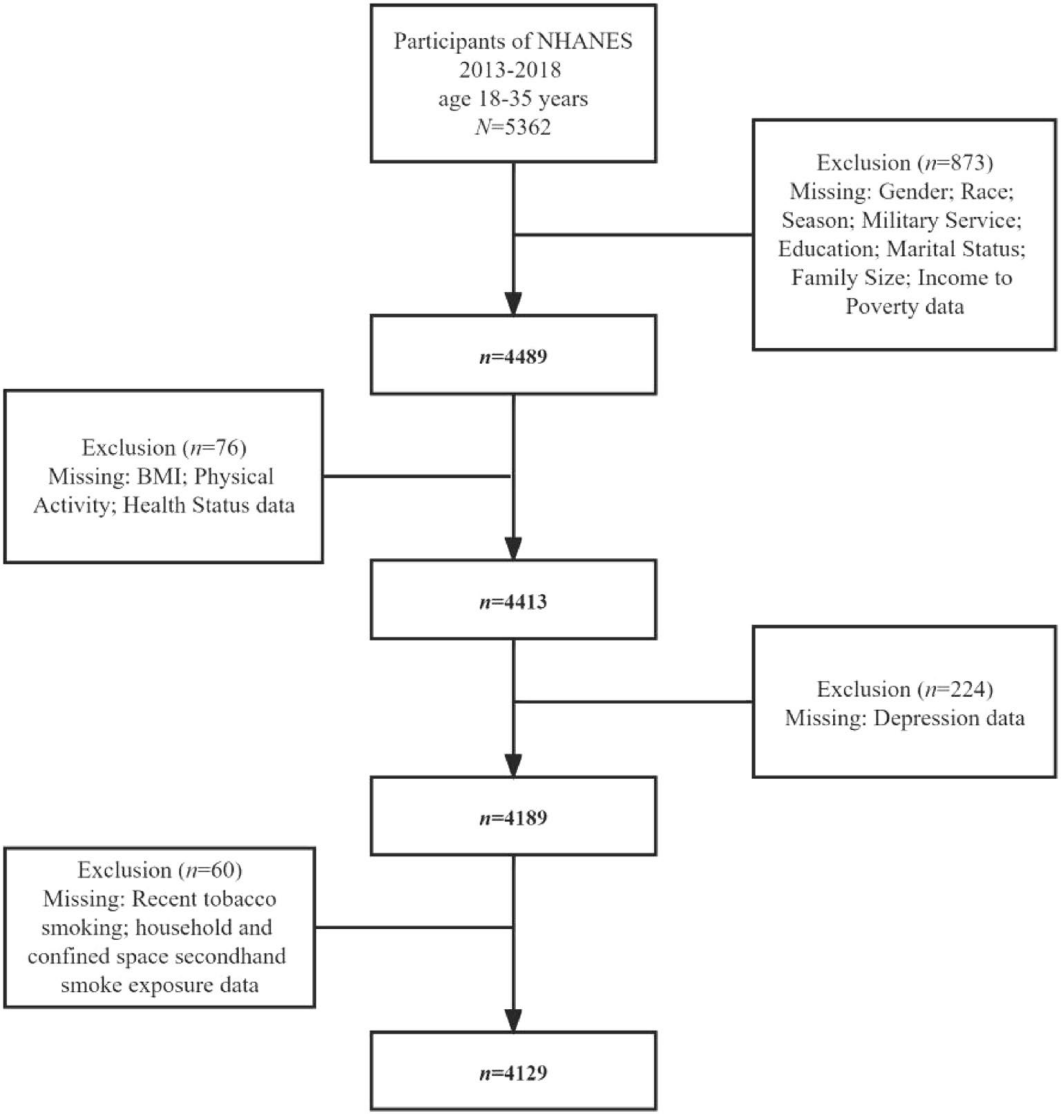
Flow chart of subject selection.

The population for this study was obtained from the National Health and Nutrition Examination Survey (NHANES). The NHANES is a population based cross-sectional survey designed to collect information on the health and nutrition of the U.S. household population. The survey is conducted on a two-year cycle and consists of both household interviews and health assessments. NHANES protocols and secondary analyses of the data were approved by the National Center for Health Statistics Research (NCHS) Ethics Review Board, and all adult participants provided written notification of consent. NHANES used a stratified multi-stage sampling design to obtain a representative sample of U.S. residents. The sampling plan consisted of four stages: selection of primary sampling units, counties or neighboring group counties; selection of units within counties; selection of dwelling units and selection of sample persons within dwelling units. In this study, participants aged 18–35 years in NHANES 2013–2018 were included as "young adults"^12,13^. The sampling plan was as follows: First, data on demographic characteristics, body measurements, current health status, depression, recent tobacco smoking, and secondhand smoke exposure specifically for the target population of this study from NHANES were obtained. Second, participants who had invalid demographic information (missing data) were excluded. Afterwards, participants who had invalid data (missing data) regarding body measurements and health status were excluded. Lastly, participants who had invalid data (including missing data, responding "don't know," and refusal to answer the question) for both the independent variables (recent tobacco smoking and secondhand smoke exposure) and the dependent variable (depression) of the study were excluded. The total sample size included in this study was 4129. Additional details regarding the study design, sampling and exclusion criteria are presented in the flow chart below. (See Fig. 1).

### Depressive symptom assessment

Depressive symptoms in this study were assessed by the Patient Health Questionnaire (PHQ-9), a 9-item depression screening instrument used to assess the frequency with which participants experienced depressive symptoms in the past 2 weeks. The items in the questionnaire were asked at the Mobile Examination Center (MEC) by trained interviewers using a computer-assisted personal interview (CAPI) system, which was programmed with built-in consistency checks to reduce data entry errors, as part of the MEC interview. For each item, points ranging from 0 to 3 represent the response categories of “not at all,” “a few days,” “more than half the days,” and “almost every day,” respectively. Those with complete responses to the symptom questions could calculate a total score ranging from 0 to 27. Those with scores below 5 were considered to have no depressive symptoms; those with scores 5–9 were considered to have mild depressive symptoms; those with scores 9–19 were considered to have moderate depressive symptoms; and those with scores over 19 were considered to have severe depressive symptoms^14^. In the present study, participants scored 0–4 were classified as the “Non-depressive Group” and scored of 5–27 were classified as the “Depressive Group”.

### Recent tobacco smoking assessment

The NHANES 2013–18 “Smoking-Recent Tobacco Use (SMQRTU_J)” datasets were used for Recent Tobacco Smoking Assessment. The SMQRTU datasets provided detailed information on the use of cigarettes, pipes, cigars, and other forms of tobacco, as well as nicotine replacement therapy products, within the past 5 days. The questions were asked during the MEC interview. For adults 18 years and older, questions were asked by trained interviewers using the CAPI system. In this study, recent tobacco smoking were assessed through question “During the past 5 days, including today, did you smoke cigarettes, pipes, cigars, little cigars or cigarillos, water pipes, hookahs, or e-cigarettes?”.

### Household secondhand smoke exposure assessment

The NHANES 2013–18 “Smoking-Household Smokers (SMQFAM_J)” datasets were used in this study for household secondhand smoke exposure assessment. The SMQFAM datasets provided information about tobacco smoking among persons living in the home. The two questions in the datasets included two aspects: (1) whether the participant lived with a smoker, and (2) whether someone smoked in the participant's home. In this study, participants who answered positive to both of these questions were considered to have household secondhand smoke exposure.

### Confined space secondhand smoke exposure assessment

Confined space secondhand smoke exposure was assessed using the Secondhand Smoke Exposure Questionnaire (SSEQ) developed for this study. The internal consistency reliability of the SSEQ is acceptable and the validity is good, for more information please refer to the second paragraph of the results section (see Tables 2 and 3). The items in SSEQ were extracted from the NHANES 2013–18 “Smoking-Secondhand Smoke Exposure (SMQSHS_J)” datasets, which provided information on potential exposure to cigarette and tobacco smoke from other people in a variety of indoor environments in the past 7 days. Questions in the datasets were asked as part of a questionnaire for the sample population, at home, using CAPI system for people aged 18 years and older. All confined space exposures were considered in the six items of the SSEQ, including at job, in restaurant, in bar, in car, in another person’s home, and in other indoors. For each item, points of 0 and 1 represent “do not have this type of exposure” and “have had this type of exposure,” respectively. In this study, participants reported secondhand smoke exposure in at least one of the six items (SSEQ score ≥ 1) were considered to have confined space secondhand smoke exposure. Additionally, the six items of the SSEQ were also assessed as independent predictors.

### Covariates

Covariates that may be associated with depressive symptoms were included in the logistic regression models to ensure accuracy. The covariates considered in this study encompassed demographic characteristics, body mass index (BMI), moderate-to-vigorous physical activity (MVPA), and current health status. Demographic covariates included age, gender (male/female), race (Hispanic/non-Hispanic White/non-Hispanic Black/ non-Hispanic Asian/Other), season of examination (November–April/May–October), military service (yes/no), education (below high school/high school/post-high school), marital status (cohabiting/married living alone/unmarried), household member, and Poverty Income Ratio (PIR). Among these factors, the number of people in the household could potentially influence depressive symptoms through various mechanisms. Larger families might experience greater financial stress, more frequent interpersonal conflicts, crowded living conditions, limited personal space, and overloaded family responsibilities. These reductions in social support, psychological distress, and stress resulting from larger family size could elevate the risk of experiencing depressive symptoms^15^. Therefore, household member was included as a covariate in this study. PIR was determined by dividing family income by the poverty guidelines specific to the survey year. These guidelines vary based on family size and geographic location^16^. For this study, PIR was used to establish two categories of income status: impoverished (< 1.3) and moderate income (≥ 1.3)^17^. BMI was calculated by dividing height in meters by weight in kilograms squared, measured by a trained technician using standardized equipment during the MEC physical examination. In this study, BMI was divided into four standard categories: Underweight (≤ 18.9 kg/m^2^), Normal weight (19.0–24.9 kg/m^2^), Overweight (25.0–29.9 kg/m^2^), and Obese (≥ 30.0 kg/m^2^)^18^. MVPA was evaluated through an interview based on the Global Physical Activity Questionnaire (GPAQ). In this study, MVPA was classed as “yes” and “no” based on question: “engaged in moderate or higher intensity physical activity in the past week?”^19^. Current health status was self-reported during the MEC interview and categorized into five levels: excellent, very good, good, fair, and poor. The detailed information on these covariates could be found at www.cdc.gov/nchs/nhanes/.

### Statistical analyses

R software was initially utilized to convert the original ".xpt" format data to ".xls" format. Subsequently, Microsoft Excel 2010 software were used to eliminate missing and irrelevant (participants refused or answered “don’t know”) data, calculate the mean and standard deviation of the descriptive data. The database only included adults aged 18–35 years with complete information on depressive symptoms, tobacco smoking, secondhand smoke exposure, and all covariates relevant to this study. Participants were divided into “Depressive group” and “Non-depressive group”.

For reliability assessment of the SSEQ, Cronbach's alpha analysis was employed; For validity assessment, confirmatory factor analysis (CFA) were employed. Mono-factor analysis were performed to compare differences between “Depressive group” and “Non-depressive group”: chi-square tests for categorical data, and rank-sum tests for continuous data. Post-hoc analysis were performed to compare differences between “Depressive group” and “Non-depressive group” for job, restaurant, bar, in-car, in another home, and other indoor settings secondhand smoke exposure. Binary logistic regression analysis were performed to analysis the association between recent tobacco smoking/ secondhand smoke exposures and depressive symptoms, respectively. Variables found to be statistically significant in the mono-factor analysis were included in the logistic regression analysis.

To analyze the association between recent tobacco smoking, household secondhand smoke exposure, confined space secondhand smoke exposure, and depressive symptoms. Recent tobacco smoking, household secondhand smoke exposure, and confined space secondhand smoke exposure were treated as independent variables (0 = no, 1 = yes), and depressive symptoms was treated as the dependent variable (0 = non-depressive, 1 = depressive). To ensure accuracy, the following models were developed : Model I: Original model without adjusting for any variables; Model II: Adjusting for covariates (gender, race, household member, PIR, BMI, current health status); Model III and IV: Adjusting for the variables in the previous model plus one independent variable (e.g., adjusting “household secondhand smoke exposure” in Model III, and adjusting “confined space secondhand smoke exposure” in Model IV, when analyzing the association between recent tobacco smoking and depression).

To analyze the association between restaurant, bar, in-car, in another home, other indoor settings in terms of secondhand smoke exposure in and depressive symptoms, the five types of exposure were treated as independent variables (0 = no, 1 = yes), and depressive symptoms was treated as the dependent variable (0 = non-depressive, 1 = depressive). To ensure accuracy, the following models were developed: Model V: Original model without adjusting for any variables; Model VI: Adjusting for covariates; Model VII: Adjusted for the variables in Model VI plus recent tobacco smoking; Model VIII: Adjusted for the variables in Model VII plus household secondhand smoke exposure; Model IX: Adjusted for the variables in Model VIII plus the rest four independent variables (e.g., adjusting for bar, in-car, in another home, other indoor settings secondhand smoke exposure in Model IX, when analyzing the association between restaurants secondhand smoke exposure in and depressive symptoms).

All data analyses were performed using the Statistical Package for Social Sciences (SPSS) version 28.0. P values less than 0.05 were considered statistically significant (two-tailed test).

### Ethics approval and consent to participate

All procedures performed in the study were in accordance with the Declaration of Helsinki. The study protocols for NHANES were approved by the National Center for Health Statistics (NCHS) Research Ethics Review Board (Protocol#2013–1, Protocol#2015–1, Protocol#2017–1). All adult participants provided written notification of consent before participating in the study.

## Results

### Demographic characteristics

This study involved a total of 4129 participants, with a mean age of 26.11 ± 5.39 years at the time of examination, including 2021 males and 2108 females. Significant statistical differences were found in gender, race, household member, PIR, BMI, current health status, recent tobacco smoke, household secondhand smoke exposure, and confined space secondhand smoke exposure between the depressive group and the non-depressive group (*P* < 0.001). However, no significant differences were observed in age (*P* = 0.068), season of exam (*P* = 0.619), military service (*P* = 0.947), education (*P* = 0.191), marital status (*P* = 0.052), and MVPA (*P* = 0.086). (See Table 1).

**Table 1:** Demographic characteristics of NHANES 2013–18 young adults aged 18–35 years, by depressive symptom.

Characteristics, n%	Sample capacity		Depressive		Non-depressive		Test statistics	*P* value
	N = 4129		n = 966	(23.40)	n = 3163	(76.60)		
Age	26.11 ± 5.39		25.75 ± 5.41		26.22 ± 5.38		− 0.513^b^	0.608
Gender							14.523^a^	< 0.001
Male	2021	(48.95)	421	(43.58)	1600	(50.58)		
Female	2108	(51.05)	545	(56.42)	1563	(49.42)		
Race							10.626^a^	0.031
Hispanic	1087	(26.33)	257	(26.60)	830	(26.24)		
Non-Hispanic White	1423	(34.46)	339	(35.09)	1084	(34.27)		
Non-Hispanic Black	861	(20.85)	200	(20.70)	661	(20.90)		
Non-Hispanic Asian	518	(12.55)	99	(10.25)	419	(13.25)		
Other	240	(5.81)	71	(7.35)	169	(5.34)		
Season of exam							0.247^a^	0.619
November–April	2057	(49.82)	488	(50.52)	1569	(49.60)		
May–October	2072	(50.18)	478	(49.48)	1594	(50.40)		
Military service							0.001^a^	0.947
Yes	102	(2.47)	24	(2.48)	78	(2.47)		
No	4027	(97.53)	942	(97.52)	3085	(97.53)		
Education							3.310^a^	0.191
Below high school	723	(17.51)	181	(18.74)	542	(17.14)		
High school	1122	(27.17)	275	(28.47)	847	(26.78)		
Post high school	2284	(55.32)	510	(52.80)	1774	(56.09)		
Marital statues							5.899^a^	0.052
Cohabitation	1745	(42.26)	376	(38.92)	1369	(43.28)		
Married living alone	182	(4.41)	43	(4.45)	139	(4.39)		
Not married	2202	(53.33)	547	(56.63)	1655	(52.32)		
Household member(s)							− 0.201^b^	0.028
One	776	(18.79)	185	(19.15)	591	(18.68)		
Two	542	(13.13)	138	(14.29)	404	(12.77)		
Three	785	(19.01)	172	(17.81)	613	(19.38)		
Four	794	(19.23)	183	(18.94)	611	(19.32)		
Five	626	(15.16)	138	(14.29)	488	(15.43)		
Six and above	606	(14.68)	150	(15.53)	446	(14.10)		
PIR							22.533^a^	< 0.001
Impoverished	1524	(36.91)	405	(41.93)	1119	(35.38)		
Moderate income	2605	(63.09)	516	(53.42)	2044	(64.62)		
BMI							10.005^a^	0.019
Underweight	184	(4.46)	45	(4.66)	139	(4.39)		
Normal weight	1449	(35.09)	313	(32.40)	1136	(35.92)		
Overweight	1099	(26.62)	242	(25.05)	857	(27.09)		
Obese	1397	(33.83)	366	(37.89)	1031	(32.60)		
MVPA							2.940^a^	0.086
Yes	3516	(85.15)	806	(83.44)	2710	(85.68)		
No	613	(14.85)	160	(16.56)	453	(14.32)		
Current health status							229.111^a^	< 0.001
Excellent	449	(10.87)	48	(4.97)	401	(12.68)		
Very good	1298	(31.44)	207	(21.43)	1091	(34.49)		
Good	1710	(41.41)	427	(44.20)	1283	(40.56)		
Fair	611	(14.80)	247	(25.57)	364	(11.51)		
Poor	61	(1.48)	37	(3.83)	24	(0.76)		
Recent tobacco smoke							83.493^a^	< 0.001
Yes	1152	(27.90)	381	(39.44)	771	(24.38)		
No	2977	(72.10)	585	(60.56)	2392	(75.62)		
Household secondhand smoke exposure							51.559^a^	< 0.001
Yes	1384	(33.52)	416	(43.06)	968	(30.60)		
No	2745	(66.48)	550	(56.94)	2195	(69.40)		
Confined space secondhand smoke exposure							64.200^a^	< 0.001
Yes	1509	(36.55)	458	(47.41)	1051	(33.23)		
No	2620	(63.45)	508	(52.59)	2112	(66.77)		

*BMI* body mass index, *MVPA* moderate-to-vigorous physical activity.

^a^Chi-square test.

^b^Rank-sum test; Depression: PHQ-9 score ≥ 5, non-Depression: PHQ-9 score < 5.

### Reliability and validity analysis

The results of the Cronbach's s α reliability analysis demonstrated that the SSEQ exhibited satisfactory internal consistency. The total Cronbach's s α coefficient of the SSEQ was 0.676, with a standardized Cronbach's s α coefficient of 0.674. Additionally, the item removed Cronbach's s α coefficients for each item were as follows: 0.657, 0.666, 0.660, 0.599, 0.611, and 0.605. These results indicate that the SSEQ demonstrates acceptable internal consistency, as it exceeds the recommended threshold of 0.6^20^. (See Table 2).

**Table 2:** Internal consistency of the secondhand smoke exposure questionnaire in this study.

Item	CITC	Item removed α coefficient	Cronbach’s α coefficient
Last 7-d at job someone smoked indoors	0.335	0.657	0.676
Last 7-d at rest someone smoked indoors	0.305	0.666	
Last 7-d in bar someone smoked indoors	0.344	0.660	
Last 7-d someone smoked in car	0.500	0.599	
Last 7-d in home someone smoked indoor	0.473	0.611	
Last 7-d in other indoor someone smoked	0.942	0.605	
Standardized Cronbach’s α coefficient			0.674

*CITC* corrected item total correlation.

The results of CFA indicated that the KOM value of the SSEQ was 0.702, and Bartlett's spherical test approximated χ^2^ = 222.023 (*P* < 0.001). Two common factors with eigenvalues > 1 could be extracted from SSEQ, and the cumulative variance contribution of the two common factors extracted, which consisted of 4 and 2 items respectively, was 58.882%. The communalities for each item of the SSEQ were 0.311, 0.714, 0.692, 0.624, 0.638, and 0.509, respectively. The results of the CFA indicate that the SSEQ had good structural validity. The communalities for item "Last 7-d at job someone smoked indoors" was less than 0.4, indicating that this item is not sufficient to convey the valid information it covers^21^. However, we believe that this item has a maximum correlation with theoretical relevance and perceived relevance by the participants of the SSEQ as a whole^22^. In addition, the item contributes to the fit of the overall model with its relatively significant factor loadings and unique contribution to the overall variance explained by the model^23^. Thus, this item was retained for further analysis. (See Table 3).

**Table 3:** The validity of the secondhand smoke exposure questionnaire in this study.

Item	Factor loading capacity		Communalities
	Factor 1	Factor 2	
Last 7-d at job someone smoked indoors	0.532	− 0.167	0.311
Last 7-d at rest someone smoked indoors	0.461	0.708	0.714
Last 7-d in bar someone smoked indoors	0.539	0.633	0.692
Last 7-d someone smoked in car	0.725	− 0.315	0.624
Last 7-d in home someone smoked indoors	0.708	− 0.427	0.683
Last 7-d in other indoor someone smoked	0.710	− 0.069	0.509
Eigenvalue (before)	2.316	1.127	
Variance interpretation (before)	28.607%	20.275%	
Cumulative variance interpretation (before)	38.607%	58.822%	
Eigenvalue (after)	2.056	1.477	
Variance interpretation (after)	34.265%	34.265%	
Cumulative variance interpretation (after)	24.617%	58.882%	
KMO	0.702		
Bartlett's spherical	222.023		
df	15		
*P*	< 0.001		

*KMO* Kaiser–Meyer–Olkin Value; (before): Before Rotation; (after): After Rotation.

### Post-hoc analysis

Post-hoc analyses revealed significant differences in restaurant (*P* = 0.003), bar (*P* = 0.013), in-car (*P* < 0.001), in another home (*P* < 0.001), and other indoor settings (*P* < 0.001) in terms of secondhand smoke exposure between the depressive group and the non-depressive group. However, no significant difference was observed in job secondhand smoke exposure (*P* = 0.997). (See Table 4).

**Table 4:** Secondhand smoking exposure settings of NHANES 2013–18 adults aged 18–35 by depressive symptom.

Predictors	Sample capacity		Depression		Non-depression		Exp(B)	95% CI	*P*
	N = 4129		n = 966		n = 3163				
At job							1.001	0.779–1.285	0.997
Yes	376	(9.11)	88	(9.11)	288	(9.11)			
No	3752	(90.87)	878	(90.89)	2875	(90.89)			
In restaurant							1.800	1.223–2.651	0.003
Yes	117	(2.83)	41	(4.24)	76	(2.40)			
No	4012	(97.17)	925	(95.76)	3087	(97.60)			
In bar							1.447	1.803–1.934	0.013
Yes	232	(5.62)	70	(7.25)	162	(5.12)			
No	3897	(94.38)	896	(92.75)	3001	(94.88)			
In car							2.001	1.704–2.349	< 0.001
Yes	941	(22.79)	318	(32.92)	623	(19.70)			
No	3188	(77.21)	648	(67.08)	2540	(80.30)			
In another home							1.780	1.462–2.168	< 0.001
Yes	531	(12.86)	177	(18.32)	354	(11.19)			
No	3598	(87.14)	789	(81.68)	2809	(88.81)			
In other indoors							1.629	1.266–2.097	< 0.001
Yes	303	(7.34)	98	(10.14)	205	(6.48)			
No	3826	(92.66)	868	(89.86)	2958	(93.52)			

### Logistic regression analyses

#### Associations of smoking related exposures and depressive symptoms

In this part of the analysis, gender, race, household member(s), PIR, BMI, and current health status were included as covariates in the regression models. For accuracy in the results, independent variables other than the current predictor were individually adjusted. Binary logistic regression analyses were conducted separately to assess the association between recent tobacco smoking, household secondhand smoke exposure, and confined space secondhand smoke exposure with depressive symptoms in young adults.

Recent tobacco smoking exposure was found to be significantly associated with depressive symptoms in the original model (Model I: OR = 2.201, 95% CI 1.735–2.353), the covariate-adjusted model (Model II: OR = 1.904, 95% CI 1.621–2.237), and the final model (Model IV: OR = 1.593, 95% CI 1.318–1.926). Household secondhand smoke exposure was found to be significantly associated with depressive symptoms in the original model (Model I: OR = 1.715, 95% CI 1.479–1.989) and the covariate-adjusted model (Model II: OR = 1.339, 95% CI 1.339–1.827). However, this association did not reach statistical significance in the final model (Model IV: *P* = 0.185). Confined space secondhand smoke exposure was found to be significantly associated with depressive symptoms in the original model (Model I: OR = 1.812, 95% CI 1.565–2.097), the covariate-adjusted model (Model II: OR = 1.674, 95% CI 1.435–1.952), and the final model (Model IV: OR = 1.399, 95% CI 1.185–1.651).

The results suggest that recent tobacco smoking and confined space secondhand smoke exposure may act as potential risk factors for depressive symptoms in young adults, potentially increasing the risk by 31.8 ~ 92.6% and 18.5 ~ 65.1%, respectively. (See Table 5).

**Table 5:** Weighted association of recent tobacco smoking, household/confined space secondhand smoke exposure and depressive symptom in NHANES 2013–18 adults aged 18–35 years.

Predictors	*b*	SE	*Wald*	*P*	OR (95% CI)
Recent tobacco smoking					
Model I^c^	0.703	0.078	81.788	< 0.001	2.201 (1.735–2.353)
Model II^d^	0.644	0.082	61.437	< 0.001	1.904 (1.621–2.237)
Model III^f^	0.547	0.095	33.438	< 0.001	1.728 (1.436–2.080)
Model IV^g^	0.466	0.097	23.167	< 0.001	1.593 (1.318–1.926)
Household secondhand smoke exposure					
Model I^c^	0.539	0.076	50.963	< 0.001	1.715 (1.479–1.989)
Model II^d^	0.447	0.079	31.768	< 0.001	1.339 (1.339–1.827)
Model III^e^	0.190	0.092	4.268	0.038	1.209 (1.010–1.448)
Model IV^g^	0.124	0.094	1.760	0.185	1.132 (0.942–1.360)
Confined space secondhand smoke exposure					
Model I^c^	0.594	0.075	63.328	< 0.001	1.812 (1.565–2.097)
Model II^d^	0.515	0.078	43.162	< 0.001	1.674 (1.435–1.952)
Model III^e^	0.356	0.083	18.261	< 0.001	1.427 (1.212–1.680)
Model IV^f^	0.336	0.085	15.730	< 0.001	1.399 (1.185–1.651)

^c^Original model without adjusting for any variables.

^d^Adjusting for covariates.

^e^Adjusting for variables in the last model plus recent tobacco smoking.

^f^Adjusting for variables in the last model plus household secondhand smoke exposure.

^g^Adjusting for variables in the last model plus confined space secondhand smoke exposure; Same as below.

#### Associations of secondhand smoke exposures in different indoor settings and depressive symptoms

In this part of the analysis, gender, race, household member(s), PIR, BMI, and current health status were included as covariates in the regression models. For accuracy in the results, independent variables other than the current predictor were individually adjusted. Binary logistic regression analyses were conducted separately to assess the association between restaurant, bar, in-car, in another home, other indoor settings in terms of secondhand smoke exposure with depressive symptoms in young adults.

Restaurant exposure was found to be significantly associated with depressive symptoms in the original model (Model V: OR = 1.800, 95% CI 1.223–2.561), the covariate-adjusted model (Model VI: OR = 1.904, 95% CI 1.621–2.237), and the final model (Model IX: OR = 1.732, 95% CI 1.120–2.678). Bar exposure was found to be significantly associated with depressive symptoms in the original model (Model V: OR = 1.447, 95% CI 1.083–1.934), the covariate-adjusted model (Model VI: OR = 1.403 (95% CI 1.036–1.899). However, this association did not reach statistical significance in the final model (Model IX: *P* = 0.934). In-car exposure was found to be significantly associated with depressive symptoms in the original model (Model V: OR = 2.001, 95% CI 1.704–2.349), the covariate-adjusted model (Model VI: OR = 1.843, 95% CI 1.557–2.182), and the final model (Model IX: OR = 1.350, 95% CI 1.102–1.652). Exposure in another home was found to be significantly associated with depressive symptoms in the original model (Model V: OR = 1.780, 95% CI 1.462–2.168), the covariate-adjusted model (Model VI: OR = 1.625, 95% CI 1.322–1.998). However, this association did not reach statistical significance in the final model (Model IX: *P* = 0.202). Exposure in other indoor settings was found to be significantly associated with depressive symptoms in the original model (Model V: OR = 1.629, 95% CI 1.266–2.097), the covariate-adjusted model (Model VI: OR = 1.619, 95% CI 1.243–2.100). However, this association did not reach statistical significance in the final model (Model IX: *P* = 0.148).

The results suggest that restaurant and in-car secondhand smoke exposure may act as potential risk factors for depressive symptoms in young adults, potentially increasing the risk by 12.0 ~ 167.8% and 10.2 ~ 65.2%, respectively. (See Table 6).

**Table 6:** Weighted association of secondhand smoke exposure in different settings and depressive symptom in NHANES 2013–18 adults aged 18–35 years.

	*b*	SE	*Wald*	*P*	OR (95% CI)
In restaurant					
Model V^c^	0.588	0.197	8.876	0.003	1.800 (1.223–2.561)
Model VI^d^	0.649	0.207	9.811	0.002	1.913 (1.275–2.872)
Model VII^e^	0.664	0.210	10.025	0.002	1.943 (1.288–2.932)
Model VIII^f^	0.648	0.210	9.537	0.002	1.912 (1.267–2.884)
Model IX^h^	0.549	0.222	6.104	0.013	1.732 (1.120–2.678)
In bar					
Model V^c^	0.370	0.148	6.237	0.013	1.447 (1.083–1.934)
Model VI^d^	0.338	0.155	4.780	0.029	1.403 (1.036–1.899)
Model VII^e^	0.218	0.157	1.919	0.166	1.243 (0.914–1.692)
Model VIII^f^	0.209	0.157	1.775	0.183	1.223 (0.906–1.678)
Model IX^h^	-0.012	0.168	0.005	0.943	0.998 (0.711–1.373)
In car					
Model V^c^	0.694	0.082	71.932	< 0.001	2.001 (1.704–2.349)
Model VI^d^	0.611	0.086	50.371	< 0.001	1.843 (1.557–2.182)
Model VII^e^	0.409	0.094	18.927	< 0.001	1.505 (1.252–1.809)
Model VIII^f^	0.384	0.097	15.741	< 0.001	1.468 (1.214–1.774)
Model IX^h^	0.300	0.103	8.442	0.004	1.350 (1.102–1.652)
In another home					
Model V^c^	0.577	0.100	32.932	< 0.001	1.780 (1.462–2.168)
Model VI^d^	0.486	0.105	21.220	< 0.001	1.625 (1.322–1.998)
Model VII^e^	0.321	0.109	8.745	0.003	1.378 (1.114–1.705)
Model VIII^f^	0.306	0.109	7.924	0.005	1.359 (1.098–1.682)
Model IX^h^	0.150	0.117	1.631	0.202	1.161 (0.923–1.461)
In other indoors					
Model V^c^	0.488	0.129	14.372	< 0.001	1.629 (1.266–2.097)
Model VI^d^	0.482	0.135	12.734	< 0.001	1.619 (1.243–2.110)
Model VII^e^	0.386	0.137	7.967	0.005	1.470 (1.125–1.922)
Model VIII^f^	0.366	0.137	7.152	0.007	1.442 (1.103–1.887)
Model IX^h^	0.207	0.143	2.090	0.148	1.230 (0.929–1.629)

^h^Adjusting for variables in the last model plus the rest four independent variables.

## Discussion

### Recent tobacco smoking and depressive symptom

The results of this study suggest that tobacco smoking in the last five days is significantly associated with depressive symptoms in young adults. Smoking has been shown to have numerous adverse effects on mental health, including an increased risk of depression^24^. Nicotine, the main component of tobacco smoke, can rapidly affect the levels of neurotransmitters in the brain, leading to changes in mood and behavior^25^. In addition, acute smoking can lead to oxidative stress and inflammation, which are also associated with depression^26^. In fact, it has been found previously that recent smoking is significantly associated with a higher prevalence of depressive symptoms in young adults^27^. Smoking behavior may also be influenced by a range of personal and social factors. For example, individuals experiencing stress, anxiety, or low mood may be more likely to smoke as a way to cope with negative emotions^28^. In addition, smoking may be perceived as a way to fit into a social group or as a means of rebelling against authority^29^. However, the social stigma associated with smoking may also lead to feelings of shame and guilt, which may exacerbate depressive symptoms^30^, especially for adolescents who have just started smoking.

### Household exposure and depressive symptom

The results of this study suggest that household secondhand smoke exposure is not significantly associated with depressive symptom in young adults after adjusting for the effects of active smoking as well as other confined space secondhand smoke exposures. Previous research has found that household secondhand smoke exposure is associated with a variety of adverse health outcomes, including respiratory and cardiovascular disease^31^. However, the association of household exposure and depression in young adults is not clear. This may be because exposure to secondhand smoke is typically lower in the home than in other settings^32^, such as restaurants and cars. In addition, the effects of secondhand smoke on mental health may be mediated by other factors, such as stress and social support, rather than direct exposure^33^. The lack of association between household exposure and depression in young adults may also be explained by the social context of exposure. Secondhand smoke exposure in the home is often the result of living with family members who smoke, which may be perceived as a more acceptable behavior and less stigmatizing than smoking in public^34^. As a result, individuals may be more accepting of this exposure and less likely to perceive it as a risk to their health. In addition, the sense of connection and support within the family may also offset the multiple negative effects of secondhand smoke exposure on mental health. For example, smokers who smoke at home tend to avoid non-smoking household members, especially the vulnerable children or adolescent members. Overall, both biomedical and psycho-social perspectives may explain the lack of association between household secondhand smoke exposure and depression in young adults. Although household secondhand smoke exposure is associated with a variety of adverse health outcomes, it may not be relevant for mental health.

### Confined space exposure and depressive symptom

The results suggest that across all confined space settings, some secondhand smoke exposures (restaurant, in-car) may increase the risk of depressive symptom in young adults, but not the others (bar, household and in another home). It has been suggested that the smoking bans enacted in recent years in workplaces and other public places may expose fewer people to secondhand smoke in these environments than in the past. In contrast, restrictions on smoking in car and restaurant may be less prevalent. This could explain why exposure in some environments may be more likely to become important risk factors for depressive symptoms^35^. However, this explanation does not truly include all results of our study. We attribute our results to the fact that participants had different expectations about the likelihood of exposure to secondhand smoke in different settings. The expectancy-value theory suggested that human behavior is influenced by the value a person places on a particular outcome and their beliefs about the likelihood of that outcome occurring^36^. This theory could be particularly relevant when explaining the association of secondhand smoke exposure and depressive symptoms.

Exposures in restaurant and car are potential risk factors for depressive symptom, this may be because exposure in these settings is often not expected, and thus may increase the perceived likelihood of adverse health outcomes. For example, people always want to eat peacefully in a restaurant and rest peacefully in a car, and don't expect to be disturbed by secondhand smoke. In contrast, exposures at job, bar, household and other’s home had no significant effect. This may be because exposures in these settings is often expected and therefore less likely to be perceived as a risk. For example, people have long in their perception associated bars, workplace, or the specific family member with smoke^37^. These different psychological activities lead to the possibility that people may be motivated to avoid secondhand smoke in some settings, but not in others.

However, the reality is that secondhand smoke exposure is often difficult to avoid and people are often exposed by the time they discover it^38^. When people's expectations do not align with the reality of their situation, it can lead to feelings of disappointment, frustration, and hopelessness^39^. For example, one who have expectations of a clean environment may experience feelings of disappointment and hopelessness if those expectations are not met. In addition, people's inability to respond to changes in the environment can compound these feelings of disappointment and frustration, further increasing the risk of depressive symptom^40^. For example, one who are unable to adapt to secondhand smoke exposure in an environment where smoking is theoretically not allowed may experience feelings of hopelessness and despair, further exacerbating his mental health problems.

### Limitations

This study has several limitations: most importantly (1) The questionnaire measuring exposure to secondhand smoke in confined spaces is imperfect. The internal consistency reliability of the SSEQ, although acceptable, is low; the structural validity, although not mathematically problematic, does not fully reflect the intended meaning of the items regarding exposure to secondhand smoke at job. (2) Assessments of tobacco smoking and secondhand smoke are subject to bias: The data is nationally representative, but laws on tobacco may change in different states. This may lead to biased assessments in states with strict smoking bans. (3) The assessment of recent tobacco smoking lacks clarity. Instead of “tobacco smoking in the last five days”, the use “smoking in the last 30 days” may better reflect current smoking habits and minimize bias. In future study design, we will consider avoiding these limitations: refining the SSEQ: setting up a multilevel scoring method to improve reliability, changing the way questions are asked to improve validity, and dividing questionnaire dimensions; and further explore the deeper mechanics of the effects of tobacco smoking secondhand smoke exposure on depressive symptoms in young adults based on the innovative points of this study, including but not limited to epidemiological studies, biochemical and genetic studies.

## Conclusions

Recent tobacco smoking, confined space secondhand smoke exposure, and specifically restaurant and in-car exposure are associated with a greater risk of depressive symptom among U.S. young adults. In contrast, secondhand smoke exposure at household, job, bar, and in another people's home, as well as exposure to secondhand smoke in indoor settings other than those listed above, were not significantly associated with depressive symptom among U.S. young adults.

## Data Availability

The datasets generated and/or analyzed during the current study are available in the [NHANES] repository, [NHANES Questionnaires, Datasets, and Related Documentation (cdc.gov)]. Raw data supporting the obtained results are available at the corresponding author.

## Abbreviations

BMI: Body mass index
CFA: Confirmatory factor analysis
CAPI: Computer-assisted personal interview
CDC: Centers for disease control and prevention
COVID-19: Corona virus disease 2019
CI: Confidence interval
GPAQ: Global physical activity questionnaire
MEC: Mobile examination center
MVPA: Moderate-to-vigorous physical activity
NHANES: National health and nutritional examination survey
NCHS: National center for health statistics
OR: Odds ratio
PHQ-9: The patient health questionnaire
P: P-value
SSEQ: Secondhand smoke exposure questionnaire
U.S: The United States of America
WHO: World Health Organization
